# Homolactic fermentation from glucose and cellobiose using *Bacillus subtilis*

**DOI:** 10.1186/1475-2859-8-23

**Published:** 2009-04-21

**Authors:** Susana Romero-Garcia, Claudia Hernández-Bustos, Enrique Merino, Guillermo Gosset, Alfredo Martinez

**Affiliations:** 1 Departamento de Ingeniería Celular y Biocatálisis, Instituto de Biotecnología, Universidad Nacional Autónoma de México, A.P. 510-3 Cuernavaca, Mor. 62250, México; 2 Departamento de Microbiología Molecular, Instituto de Biotecnología, Universidad Nacional Autónoma de México. Cuernavaca, Morelos, México

## Abstract

**Backgroung:**

Biodegradable plastics can be made from polylactate, which is a polymer made from lactic acid. This compound can be produced from renewable resources as substrates using microorganisms. *Bacillus subtilis *is a Gram-positive bacterium recognized as a GRAS microorganism (generally regarded as safe) by the FDA. *B. subtilis *produces and secretes different kind of enzymes, such as proteases, cellulases, xylanases and amylases to utilize carbon sources more complex than the monosaccharides present in the environment. Thus, *B. subtilis *could be potentially used to hydrolyze carbohydrate polymers contained in lignocellulosic biomass to produce chemical commodities. Enzymatic hydrolysis of the cellulosic fraction of agroindustrial wastes produces cellobiose and a lower amount of glucose. Under aerobic conditions, *B. subtilis *grows using cellobiose as substrate.

**Results:**

In this study, we proved that under non-aerated conditions, *B. subtilis *ferments cellobiose to produce L-lactate with 82% of the theoretical yield, and with a specific rate of L-lactate production similar to that one obtained fermenting glucose. Under fermentative conditions in a complex media supplemented with glucose, *B. subtilis *produces L-lactate and a low amount of 2,3-butanediol. To increase the L-lactate production of this organism, we generated the *B subtilis *CH1 *alsS*^- ^strain that lacks the ability to synthesize 2,3-butanediol. Inactivation of this pathway, that competed for pyruvate availability, let a 15% increase in L-lactate yield from glucose compared with the parental strain. CH1 *alsS*^- ^fermented 5 and 10% of glucose to completion in mineral medium supplemented with yeast extract in four and nine days, respectively. CH1 *alsS*^- ^produced 105 g/L of L-lactate in this last medium supplemented with 10% of glucose. The L-lactate yield was up to 95% using mineral media, and the optical purity of L-lactate was of 99.5% since *B. subtilis *has only one gene *(lctE) *that exclusively encodes a L-lactate deshydrogenase.

**Conclusion:**

This study shows that by taking advantage of the cellobiose utilization capability and osmotic stress high resistance of *B. subtilis*, a robust process for L-lactate production can be developed.

## Background

The chemical industry has come under increasing pressure to make chemical production more eco-friendly and independent from fossil resources. The development of industrial processes based on microorganisms can importantly help to eliminate the use or generation of hazardous substances and can support the shift from the actual fossil resources dependence toward sustainable and eco-safety industrial processes [1]. One example of this eco-safety process can be found in the production of biodegradable plastics from lactic acid that involves polylactate synthesis. Physical properties and biodegradation rates of polylactate can vary depending on the D- and L-enantiomers blend [2]. In addition, lactic acid production using microorganisms eliminates the hazard substances generation and the D- and L-enantiomers can be produced in an optically pure state.

Ninety percent of current commercial lactic acid (about 72,000 ton/year) is produced from mono- and disaccharides by culture fermentation using lactic acid bacteria. It is also possible to use other renewable resources such as agroindustrial wastes as substrates to produce lactic acid, although a pretreatment to liberate simple sugars is needed [3]. Cellulose is hydrolyzed by a combination of cellobiohydrolase and endoglucanase activities with cellobiose as the primary product. The ability to utilize cellobiose is widespread among gram-positive, gram-negative and *Archeal *genera [4].

A variety of microorganisms that produce L-lactate, such as *Escherichia coli *[5,6], *Saccharomyces cerevisiae *[7], Lactobacillus sp [8,9], and lactic acid bacteria [3], have been developed for increasing their L-lactate titer, rising their L-lactate specific and volumetric productivity, rising their substrate range to include pentose sugars and disaccharides, and eliminating some of their growth requirements. Nevertheless, new biocatalysts are needed to use alternative bio-based feedstocks, like agroindustrial wastes, thus avoiding the use of fungi cellulases, exhibiting high L-lactate yields with high quiral purity.

*Bacillus subtilis *is a Gram-positive bacterium, which is a GRAS microorganism (generally regarded as safe) by the FDA. *B. subtilis *can be grown using cellobiose under aerobic conditions [10], because it has the *celRABCD *operon which encodes the EII permease and phospho-β-glucosidase proteins [4]. Under non-aerated conditions, *B. subtilis *produces L-lactate and 2,3-butanediol from glucose [11].

In this paper, we report the L-lactate synthesis from cellobiose during non-aerated cultures using *B. subtilis*. In addition, we eliminated the 2,3-butanediol production of *B. subtilis *(strain CH1 *alsS*^-^) increasing L-lactate yield by 15% compared to parental strain. CH1 *alsS*^- ^fermented 5 and 10% of glucose completely producing just optical pure L-lactate in mineral medium supplemented with yeast extract.

## Results and discussion

### *B. subtilis *produces L-lactate from cellobiose under fermentative conditions

**Figure 1:**
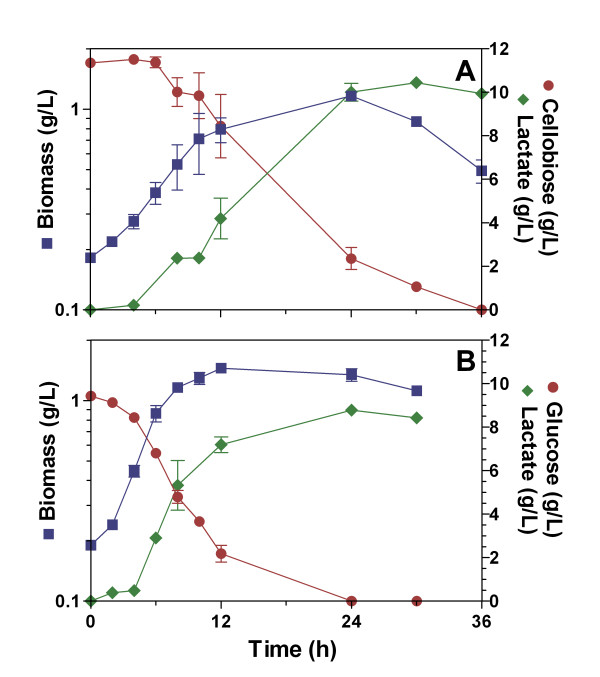
**Characterization of *Bacillus subtilis *CH1 in LB broth supplemented with 10 g/L of sugar under non-aerated conditions**. (A) Cellobiose fermentation, (B) Glucose fermentation.

**Figure 2:**
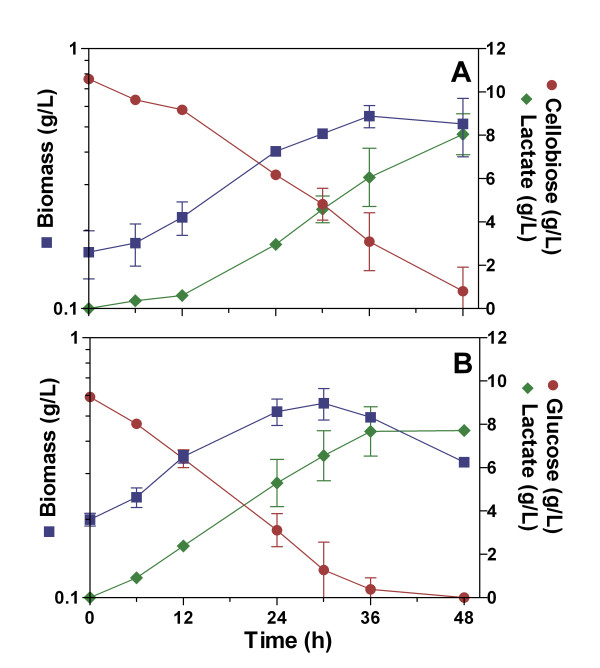
**Characterization of *Bacillus subtilis *CH1 in Mineral Medium supplemented with 10 g/L of sugar under non-aerated conditions**. (A) Cellobiose fermentation, (B) Glucose fermentation.

*B. subtilis *CH1 is a prototroph strain that was constructed to avoid the auxotrophic requirements of *B. subtilis *WB700 (see Methods section). To determine the ability of *B. subtilis *CH1 to grow using cellobiose under non-aerated conditions, the characterization of this strain using LB (Luria Bertani) and MM (Mineral Media) supplemented with cellobiose (10 g/L) was undertaken (Figs. 1A and 2A). Also, *B. subtilis *CH1 was grown in LB and MM supplemented with glucose (10 g/L) as control (Figs. 1B and 2B).

*B. subtilis *CH1 grew exponentially during the first 12 h using cellobiose or glucose in LB medium (Fig. 1A and 1B), although the specific growth rate of strain CH1 in LB medium using cellobiose diminished 2-fold in comparison with glucose (Table 1), and the maximum biomass obtained at the end of exponential growth was similar using both sugars (Table 1). The specific rate of cellobiose consumption of strain CH1 in LB medium diminished 50% in comparison with glucose; however, the specific rates of cellobiose and glucose consumption were similar in MM (Table 1).

**Table 1:** Kinetic parameters for batch cultures of *B. subtilis *CH1 grown in LB or mineral medium supplemented with glucose or cellobiose (10 g/L).

Medium	Sugar	^a ^X_MAX_ (g/L)	μ (h^-1^)	^b^Y_X/S_ (g_DCW_g_S_)	^c^q_S_ (g_S_/g_DCW _*h)	^d^Consumed sugar (g/L)
Luria	Glucose	0.67	0.30	0.26	1.17	2.6
	Cellobiose	0.61	0.13	0.23	0.60	2.9
						
Mineral	Glucose	0.32	0.04	0.05	0.77	6.1
	Cellobiose	0.30	0.04	0.06	0.66	4.8

^a^Maximum biomass obtained at the end of the exponential growth.

^b^Biomass yield from substrate during the exponential growth.

^c^Specific rate of sugar consumption during the exponential phase.

^d^Consumed sugar during the exponential growth.

The cellobiose consumption in LB medium for strain CH1 was delayed 6 h, probably due to a slight inhibition of cellobiose utilization by some media components. We suggest that during this time strain CH1 used these media components to grow and to produce L-lactate. Because *B. subtilis *could produce L-lactate from media components and from cellobiose, the L-lactate yield was higher than 100% of the theoretical if it is considered only the cellobiose cosumption (Fig. 1A and Table 2). Cellobiose utilization in MM was not delayed, supporting the hypothesis that there was inhibition by LB components (Fig. 2A).

**Table 2:** Kinetic parameters for L-lactate production for batch cultures of *B subtilis *CH1 grown in LB or mineral medium supplemented with glucose or cellobiose (10 g/L).

Medium	Sugar	^a^Y_P/S _(g_P_/g_S_)	^b^q_P _(g_P_/g_DCW _*h)	^c^q_P _(g_P_/g_DCW _*h)	^d^Q_P_ (g_p_/L*h)	^e^Final L-lactate (g/L)
Luria	Glucose	1.23	2.25	0.28	0.48	11.6
	Cellobiose	1.14	1.69	0.26	0.36	13.0
						
Mineral	Glucose	0.83	0.66	0.21	0.16	7.7
	Cellobiose	0.82	0.58	0.38	0.17	8.1

^a^L-lactate yield from sugar at the end of fermentation.

^b^Specific rate of L-lactate production during the exponential growth.

^c^Specific rate of L-lactate production during the stationary phase.

^d^Volumetric productivity of L-lactate.

^e^L-lactate produced at the end of the culture.

*B. subtilis *CH1 fermented cellobiose and glucose using mineral or complex medium, and produced L-lactate as main fermentation product. In all the culture fermentations, the L-lactate production specific rates were larger in the exponential than in the stationary phase (Table 2). The L-lactate production specific rates were similar, using cellobiose or glucose, in complex or mineral media during the stationary phase (Table 2).

Strain CH1 consumed cellobiose (10 g/L) in both LB and MM completely (Figs. 1A and 2A), and produced L-lactate with a yield of 82% of the theoretical in MM (Table 2).

It is known that some *Lactobacillus *spp use cellobiose as carbon source, but there is little information about lactic acid production from cellobiose. *L. delbrueckii *had obtained better yields and higher quantities of L-lactate from glucose in comparison with several lactic acid bacteria [3]. Recently, Adsul et al, reported a *L. delbrueckii *mutant that produced 90 g/L of lactic acid from 100 g/L of cellobiose [9]; however, they do not mention the optical purity of the product. The principal problem with lactic acid production in *Lactobacillus *spp has been the optical purity of the product which is not higher than 95% because some lactic acid bacteria have both, L- and D-lactate dehydrogenases [3,7], and the 5% of chiral impurity of the L-lactate may increase the cost associated with purification, which is very complex and expensive to solve.

Although the L-lactate yield and productivity of *L. delbrueckii *mutant are better than those from the strain tested in this report, the optical purity of the L-lactate produced by *B. subtilis *CH1 was of 99.5%, tested with an enzymatic assay that detects and quantifies exclusively the L- enantiomer of lactate. This value was compared with the total quantity of lactate measured by HPLC. Then *B. subtilis *represents an alternative to obtain optical pure L-lactate from other sugar like arabinose and polymers like cellulose. To our knowledge, there are no previous reports on cellobiose utilization producing L-lactate under fermentative conditions using *B. subtilis*.

### *B. subtilis *biomass increased when MM was supplemented with yeast extract or corn steep liquor

The growth of *B. subtilis *in mineral medium (MM) diminished compared to complex medium like LB (Table 1). To increase both, *B. subtilis *growth and L-lactate production specific rate on mineral medium, this medium was supplemented with increased quantities of yeast extract industrial grade (YE) or corn steep liquor (CSL); components that are rich in vitamins and minerals and are not expensive.

Specific growth rate of *B. subtilis *CH1 increased 6-fold or 8-fold when MM was supplemented with 8 g/L of YE or with 10 g/L of CSL, respectively. The addition of quantities larger than 8 g/L of YE or 10 g/L of CSL to mineral medium did not increase the specific growth rate of *B. subtilis *CH1, although, biomass obtained at the end of exponential phase increased in proportion with the addition of increasing quantities of YE or CSL (data not shown).

In MM supplemented with 8 g/L of YE, L-lactate yield diminished 8% in comparison with MM medium. In MM supplemented with 10 g/L of CSL, L-lactate yield increased 6% in comparison with MM medium, but CSL had small quantities of D-lactate obtaining in the batch culture a racemic mixture of L- and D-lactate.

The L-lactate production specific rates of CH1 during exponential phase of *B. subtilis *in MM supplemented with YE or CSL diminished 45% in comparison to LB, although L-lactate production specific rates during stationary phase were similar in LB or MM supplemented with YE or CSL (data not shown). Thus, MM supplemented with 8 g/L of yeast extract was chosen to assess the capability of L-lactate production.

### L-lactate yield and L-lactate production specific rate increased when butanediol synthesis was suppressed

Strain CH1 *alsS*^- ^was constructed to eliminate pyruvate competition between the L-lactate and the 2,3-butanediol biosynthetic pathways. In strain CH1 *alsS*^-^, the butanediol production was eliminated by interrupting the gene that encodes the acetolactate synthase enzyme (ALSS), which is the first enzyme of the pathway.

Strain CH1 *alsS*^- ^was characterized under non-aerated conditions using LB medium supplemented with 20 g/L of glucose, and strain CH1 was included as a control (Table 3). The elimination of the butanediol pathway in strain CH1 *alsS*^- ^affected, neither the growth specific rate, nor the maximum biomass at the end of exponential phase (Table 3).

**Table 3:** Kinetic parameters for batch cultures of *B. subtilis *CH1 and CH1 *alsS*^- ^grown in LB medium supplemented with glucose (20 g/L).

*B. subtilis *strain	^a ^X_MAX_ (g/L)	μ (h^-1^)	^b ^q_S_ (g_S_/g_DCW _*h)	^b ^q_P_ (g_P_/g_DWC _*h)	^c ^q_S_ (g_S_/g_DCW _*h)	^c ^q_P_ (g_P_/g_DCW _*h)	Butanediol (g/L)
CH1	0.66	0.34	1.79	2.29	0.52	0.47	6
CH1 *alsS*^-^	0.62	0.32	1.99	2.63	0.51	0.56	0

^a ^Maximum biomass obtained at the end of exponential growth.

q_S_: specific rate of sugar consumption.

q_P_: Specific rate of L-lactate production

^b ^During the exponential growth.

^c ^During the stationary growth.

During the exponential growth, the glucose consumption specific rate of CH1 *alsS*^- ^increased 12% compared to CH1; this behavior probably caused a 15% increase in L-lactate production specific rate of CH1 *alsS*^- ^compared to the control (Table 3). During the stationary phase, the elimination of the butanediol pathway in strain CH1 *alsS*^- ^caused more carbon to be directed to L-lactate production, thus increasing by 20% the L-lactate production specific rate of CH1 *alsS*^- ^compared to CH1 (Table 3).

L-lactate volumetric productivity of CH1 *alsS*^- ^increased 15% compared with the control. L-lactate yield of CH1 *alsS*^- ^increased 15% up of the theoretical considering just the consumed glucose, this result suggests that CH1 *alsS*^- ^produced L-lactate from glucose and components media (data not shown).

The results presented above show that the butanediol production of strain CH1 *alsS*^- ^was completely abolished, this strain produced only L-lactate in LB with high yield, productivity, and optically pure state, thus eliminating the need for purification from a racemic mixture.

### *B. subtilis *CH1 *alsS*^- ^fermented to completion 5% and 10% glucose, and produced L-lactate as the exclusive fermentation product

**Figure 3:**
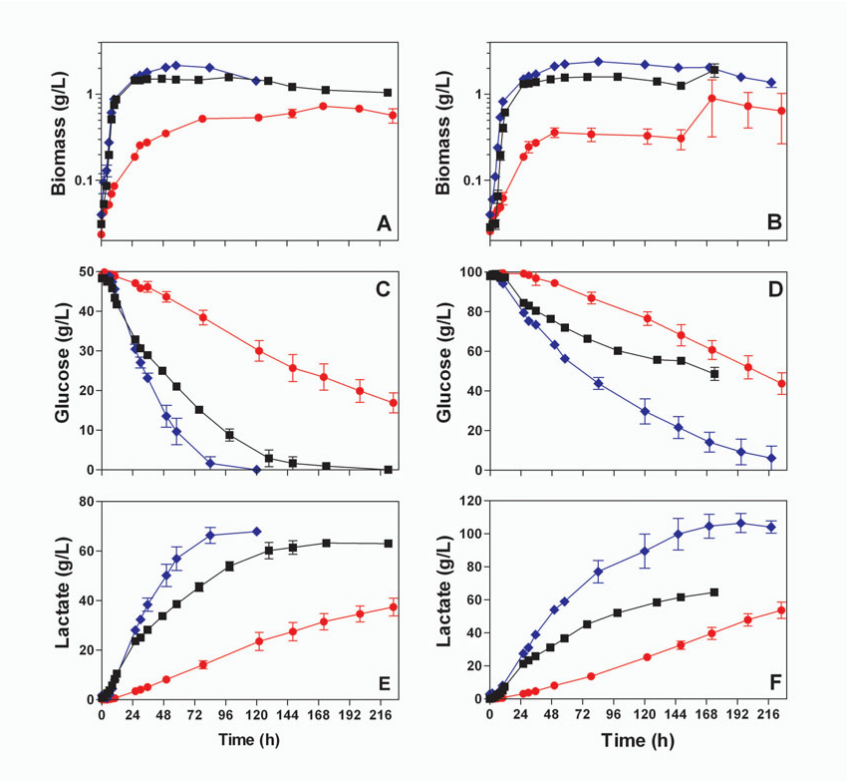
**Characterization of *B. subtilis *CH1 *alsS*^- ^using different media supplemented with 50 g/L (A, C, E) or 100 g/L (B, D, F) of glucose under non-aerated conditions**. (A, B) Biomass formation, (C, D) Glucose consumption, and (E, F) L-lactate production using different media: (square) Luria Broth, (diamond) mineral medium supplemented with 8 g/L of yeast extract, or (circle) mineral medium.

To evaluate the ability of L-lactate production of *B. subtilis *CH1 *alsS*^-^, this strain was challenged to ferment to completion 5 and 10% of glucose, just as the same high sugar concentrations of cellulosic syrups [12]. Strain CH1 *alsS*^- ^was grown under non-aerated conditions using different media: LB medium, MM, and mineral medium supplemented with 8 g/L of yeast extract (MM with YE). All of these media were supplemented with 5 or 10% of glucose. Biomass formation, glucose consumption and L-lactate production were evaluated (Fig. 3).

When LB was supplemented with 5% or 10% of glucose, the specific growth rate of CH1 *alsS*^- ^was not affected by the high glucose concentration, possibly due to addition of the non-metabolized protective osmolyte (compare data in Table 3 with Tables 1 and 3). CH1 *alsS*^- ^grew exponentially during the first 10 h, using glucose and components of the medium. During exponential growth using LB, the specific rate of glucose consumption increased by 10% when the glucose concentration was augmented from 2 to 5% or 10% (Tables 3 and 4). Throughout this time, the specific rate of L-lactate production increased 20% and 55% when CH1 *alsS*^- ^grown in LB was supplemented from 2 to 5% or 10% of glucose, respectively (Tables 3 and 5). These results suggest that two or more uptake systems are involved in the transport of glucose and that the glucose transport capacity of the cells is not reached at low glucose concentrations. A similar result has been reported for *Lactococcus lactis *[13].

**Table 4:** Kinetic parameters for batch cultures of *B. subtilis *CH1 *alsS*^- ^grown in LB and mineral medium with 8 g/L of yeast extract (MM with YE) or mineral medium (MM), supplemented with 50 or 100 g/L of glucose (Glc).

Media	^a ^X_MAX_ (g/L)	μ (h^-1^)	^b^Y_X/S_ (g_DCW_/g_S_)	^c^q_S_ (g_S_/g_DCW _*h)	^d^Consumed sugar (g/L)
50 g/L Glc					
LB	0.72	0.30	0.14	2.23	5.25
MM with YE	0.83	0.27	0.16	1.74	5.26
MM	0.16	0.06	0.06	1.09	2.91

100 g/L Glc					
LB	0.68	0.40	0.14	2.12	4.80
MM with YE	0.78	0.29	0.09	3.45	9.26
MM	0.16	0.07	0.06	1.39	3.11

^a^Maximum biomass obtained at the end of exponential growth.

^b^Biomass yield from glucose during the exponential growth.

^c^Specific rate of glucose consumption during the exponential phase.

^d^Consumed glucose during the exponential growth.

**Table 5:** Kinetic parameters for L-lactate production for batch cultures of *B subtilis *CH1 *alsS*^- ^grown in LB medium and mineral medium with 8 g/L of yeast extract (MM with YE) or mineral medium (MM), supplemented with 50 or 100 g/L of glucose (Glc).

Media	^a^Y_P/S _(g_P_/g_S_)	^b^q_P _(g_P_/g_DCW _*h)	^c^q_P_ (g_P_/g_DCW _*h)	^d^Q_P_ (g_P_/L*h)	^e^Final L-lactate (g/L)
50 g/L Glc					
LB	1.30	3.18	0.32	0.46	63.11
MM with YE	1.34	2.46	0.46	0.78	68.04
MM	0.96	1.28	0.36	0.17	37.40

100 g/L Glc					
LB	1.27	4.11	0.27	0.37	64.51
MM with YE	1.09	2.68	0.30	0.54	105.59
MM	0.92	1.33	0.67	0.24	53.69

^a^L-lactate yield from glucose at the end of fermentation.

^b^Specific rate of L-lactate production during the exponential growth.

^c^Specific rate of L-lactate production during the stationary phase.

^d^Volumetric productivity of L-lactate.

^e^L-lactate produced at the end of the stationary phase.

L-lactate production specific rates were similar during the stationary phase using LB supplemented with 5% or 10% of glucose. CH1 *alsS*^- ^consumed all the glucose and produced L-lactate, it also used some components of the rich medium increasing the L-lactate production (Fig. 3E). Although the fermentation with 10% of glucose in LB was stopped at 174 h of fermentation elapsed time, CH1 *alsS*^- ^consumed 51 g/L of glucose and produced only L-lactate (Table 5).

When MM was supplemented with 5% or 10% of glucose, the specific growth rate of CH1 *alsS*^- ^was similar in both sugar concentrations (Table 4). CH1 *alsS*^- ^grew exponentially during first 26 h, consuming 3 g/L of glucose and producing only L-lactate with yields up to 97% of the theoretical. During the exponential phase in MM, glucose consumption specific rate of CH1 *alsS*^- ^increased 25% when the glucose concentration was augmented from 5 to 10%, causing a 5% increase of the L-lactate specific production rate (Tables 4 and 5).

Throughout the stationary phase using MM, the specific rate of L-lactate production of CH1 *alsS*^- ^increased 85% when glucose concentration was increased from 5% to 10% (Table 5), although CH1 *alsS*^- ^did not finish the glucose in 9 days of elapsed time. The L-lactate yield from glucose diminished from 96% to 92% when glucose concentration was increased from 5 to 10%, because CH1 *alsS*^- ^produced small quantities of acetic acid in this condition (data not shown). Since the growth rates of the strains studied in this work were low in MM, even though it was supplemented with 1, 5 or 10% of glucose (Tables 1 and 4), the MM was supplemented with 8 g/L of yeast extract (YE) besides glucose.

When MM was supplemented with 8 g/L of yeast extract (MM with YE) and with 5% or 10% of glucose, the specific growth rate of CH1 *alsS*^- ^was similar in both sugar concentrations (Table 4). CH1 *alsS*^- ^grew exponentially during the first 10 h, using glucose and components of the medium. Throughout the exponential growth using MM with YE, the specific rate of glucose consumption had a 2-fold increase when glucose concentration was augmented from 5 to 10%, and in consequence the total consumed glucose increased 80% (Table 4). During exponential growth, the L-lactate specific production rate had a 10% increase when CH1 *alsS*^- ^grown in LB was supplemented from 5% to 10% of glucose (Table 5).

During the stationary phase using MM with YE, L-lactate specific production rate of CH1 *alsS*^- ^diminished to 30% when glucose concentration was increased from 5% to 10%. In fact, CH1 *alsS*^- ^fermented 5 or 10% of glucose using MM with YE medium to completion producing exclusively L-lactate with volumetric productivities of 0.78 and 0.54, respectively (Table 5).

The final L-lactate titer of 105 g/L for our strain CH1 *alsS*^- ^compares favorably with that from recombinant bacteria such as *L. lactis *[14] and *E. coli *SZ194 [15], and exceeds the performance of previously reported engineered biocatalysts [5,7,16].

## Conclusion

It was found that *Bacillus subtilis *can consume cellobiose under fermentative conditions and produce L-lactate with high optical purity. We established that a recombinant *B. subtilis *strain was able to produce high yields of L-lactate, using inexpensive mineral medium supplemented with 8 g/L of yeast extract. Also, the results in this study show that a robust process for L-lactate production can be developed with *B. subtilis *CH1 *alsS*^-^, taking advantage of its cellobiose utilization capability and osmotic stress high resistance.

## Methods

### Strains and Culture Media

*Bacillus subtilis *CH1 and CH1 *alsS*^- ^strains were used in all experiments. *B. subtilis *CH1 is a prototroph strain (*trp*^+^) obtained as described in [11] from *B. subtilis *WB700 strain (168, *trpC*2, Δ*nprE*, Δ*aprE*, Δ*epr*, Δ*bpf*, Δ*mpr*, Δ*nprB*, Δ*vrpE*, *Ery*^*r*^, *Lyn*^*r*^) [17]. Inactivacion of the *alsS *gene of *B. subtilis *CH1 strain to generate strain CH1 *alsS*^- ^(168, Δ*nprE*, Δ*aprE*, Δ*epr*, Δ*bpf*, Δ*mpr*, Δ*nprB*, Δ*vrpE*, Δ*alsS*, *Ery*^*r*^, *Lyn*^*r*^, *Spt*^*r*^) was performed as described in [11].

Culture media were Luria Bertani (LB) or mineral medium (MM). LB containing (per liter) 5 g yeast extract, 10 g tryptone, 5 g NaCl. LB was supplemented with different concentrations of cellobiose or glucose as indicated in Results section. MM [18] containing (per liter) 4 g (NH_4_)_2_SO_4_, 5.32 g K_2_HPO_4_, 6.4 g KH_2_PO_4_, 10 mg citric acid, 0.4 g MgSO_4_7H_2_O, 0.5 mg MnCl_2_, 4 mg CaCl_2_, and 3 mg FeSO_4_7H_2_O, pH was adjusted at 7.2 with NaOH. MM was supplemented with different concentrations of cellobiose or glucose, as indicated in Results section. MM added with glucose was supplemented with different concentrations of yeast extract (YE) or corn steep liquor (CSL), as indicated in Results section. When MM was supplemented with 5% or 10% of glucose, 2 mM of betaine (a non-metabolized protective osmolyte) [15] was added.

### Inoculum preparation and fermentation conditions

Strains from frozen vials were plated in Petri dish with solid fermentation media and incubated overnight at 37°C. Cells from plates were used to inoculate a 250 mL flask with 150 mL of the same fermentation media and incubated overnight at 37°C, 120 rpm. Erythromycin (5 μg/ml) and lyncomycin (5 μg/ml) were added to CH1 inoculum, and spectinomycin (100 μg/ml) was added to CH1 *alsS*^- ^inoculum. Cells from flasks were centrifuged and utilized as inocula at an initial optical density of 0.1 at 600 nm (OD_600_). Fermentations were performed in duplicate in minifleaker fermenters [19] containing 200 mL of fermentation culture media as indicated in Results section. Working conditions were 37°C, agitation speed of 100 rpm, and pH 7 (controlled by automatic additions of 2 or 4 N KOH). Growth was followed by measuring OD_600 _with a spectrophotometer (Lambda 11, Perking Elmer, Pomona, CA). Supernatants were separated from samples periodically taken and conserved at -20°C for later HPLC analysis.

### Analytical methods

Culture data presented are the average of two independent fermentations. OD_600 _was converted to dry cellular weight using a standard curve (1 OD_600 _= 0.35 g/L of dry cellular weight, DCW). All values of biomass, substrates and products were corrected by dilution factor due to base additions. Glucose and fermentation products in supernatants were measured by HPLC (Model 996, Waters, Millipore Company, Milford, Massachusetts, USA) equipped with a refractive index and an UV detector. Separations were carried out at 50°C, using an Aminex HPX-87H column (300 × 7.8 mm; BioRad), and 5 mM H_2_SO_4 _as mobile phase, at a flow rate of 0.5 mL/min (injection volume, 20 μl). L-lactate was measured using a biochemistry analyzer (YSI 2700 SELECT™), which contains a specific L-lactate oxidase enzyme immobilized in the YSI L-lactate membrane. The purity of L-lactate was evaluated as follows: optical purity = (L-lactate quantity determined in YSI analyzer * 100)/total lactic acid quantity determined in HPLC. In the case of MM supplemented with yeast extract or corn steep liquor, glucose was determined with the biochemistry analyzer using an YSI D-glucose membrane.

## Competing interests

The authors declare that they have no competing interests.

## Authors' contributions

SR-G participated in the design of this study, carried out cloning, construction of the homolactic fermentative strain, fermentations, data analysis and drafted the manuscript. CH-B participated in the construction of the prototroph strain, fermentations and data analysis. EM participated in writing, reviewing and to comment the manuscript. GG participated in writing, reviewing and to comment the manuscript. AM conceived the study, designed and supervised the experiments, participated in results analysis and writing of the manuscript. All authors have read and approved the manuscript.
